# Supplementary material for: Robust identification of perturbed cell types in single-cell RNA-seq data
Source: Nat Commun. 2024 Sep 1;15:7610. doi: 10.1038/s41467-024-51649-3 (PMC11366752; doi:10.1038/s41467-024-51649-3)
Supplement: Supplementary file 1 — Supplementary Information [file 41467_2024_51649_MOESM1_ESM.pdf]

Supplementary Material for "Robust identification of perturbed cell types in single-cell RNA-seq data"

A Constructing a cluster tree

This section describes how the cluster tree in Figure S24 is estimated. Initially, all annotated cell types are in a single group. Then a group is selected and split into two via the following procedure:

1. Seurat (1) is used to normalize the data and apply PCA with 50 dimensions.
2. A cell-type level pseudobulk is computed on the 50-dimensional embedding. In other words, the mean PCA score for each cell type in the group is computed.
3. k-means with $k = 2$ is applied to separate the group into two subgroups consisting of annotated cell types.

B Comparison to p-values from FMT

By default, *scDist* computes p-values using the *lmerTest* package. An alternative approach is to use the FMT method (2), a method that extends limma (3) by developing a variance shrinkage approach that be used for linear-mixed effects model. In particular, this method returns an updated degrees of freedom that can be used in a t-test for differential expression. Although it seems that this method if more relevant for the small sample-sizes of bulk RNA-seq data and is designed for log-transformed expression data, we compared this method to the p-values obtained directly from *lmerTest*. We repeated the analysis of Figure 3a that generated arbitrary groups of healthy controls with no expected biological differences. This analysis provides an estimated of the type I error rate of the hypothesis test that the distance is 0. Using the FMT method, the type I error was 0.28, well above the nominal level of 0.05 (**Fig S27**). In other words, the FMT method is not appropriate for use within the *scDist* model as it could lead to inflated p-values.

C Measuring perturbation directly with mixed models

Trabzuni and colleagues (4) introduced an approach for microarray data that in theory can be adapted to measure perturbation in single-cell RNA-seq data. Defining y_{ijg} to be the expression of gene g in cell i from patient j, the following mixed model was considered:

$$y_{ijg} = \mu + a_j + b_g + x_{ij} c_g + \varepsilon \tag{24}$$

where μ is a global intercept, a_j is a patient-specific random intercept, b_g is a gene-specific random intercept, and c_g is an additional gene-specific random intercept. Note that as before x_{ij} is the binary indicator of condition. Then the estimated variance of c_g, which we denote $\hat{\sigma}_c$, could be used as a measure of perturbation. Note that $\hat{\sigma}_c$ is similar to the $\sum_g \beta_g^2$ in the model definition of *scDist*.

Although there are parallels between this model and *scDist*, computational constraints prevent the extension of this method to single-cell RNA-seq data. Note that the expression vector in the model has length (# of cells) × (# of genes) which could be well over 10^9 on large single-cell datasets. The key innovation of *scDist* is that it avoids applying a mixed model to the entire expression vector by first reducing dimensionality with PCA.

We implemented this approach and applied it to the negative control of Figure 1 where healthy patients were divided arbitrarily into two groups. Similar to *Augur*, we see that almost all cell types have estimated variances (perturbations) above the null value of 0, falsely indicating group-level differences (Fig S28).

Moreover, this approach was significantly slower than scDist, taking nearly 40 minutes (compared to 40 seconds for scDist) on what would be considered a relatively small single-cell dataset (Fig S28).

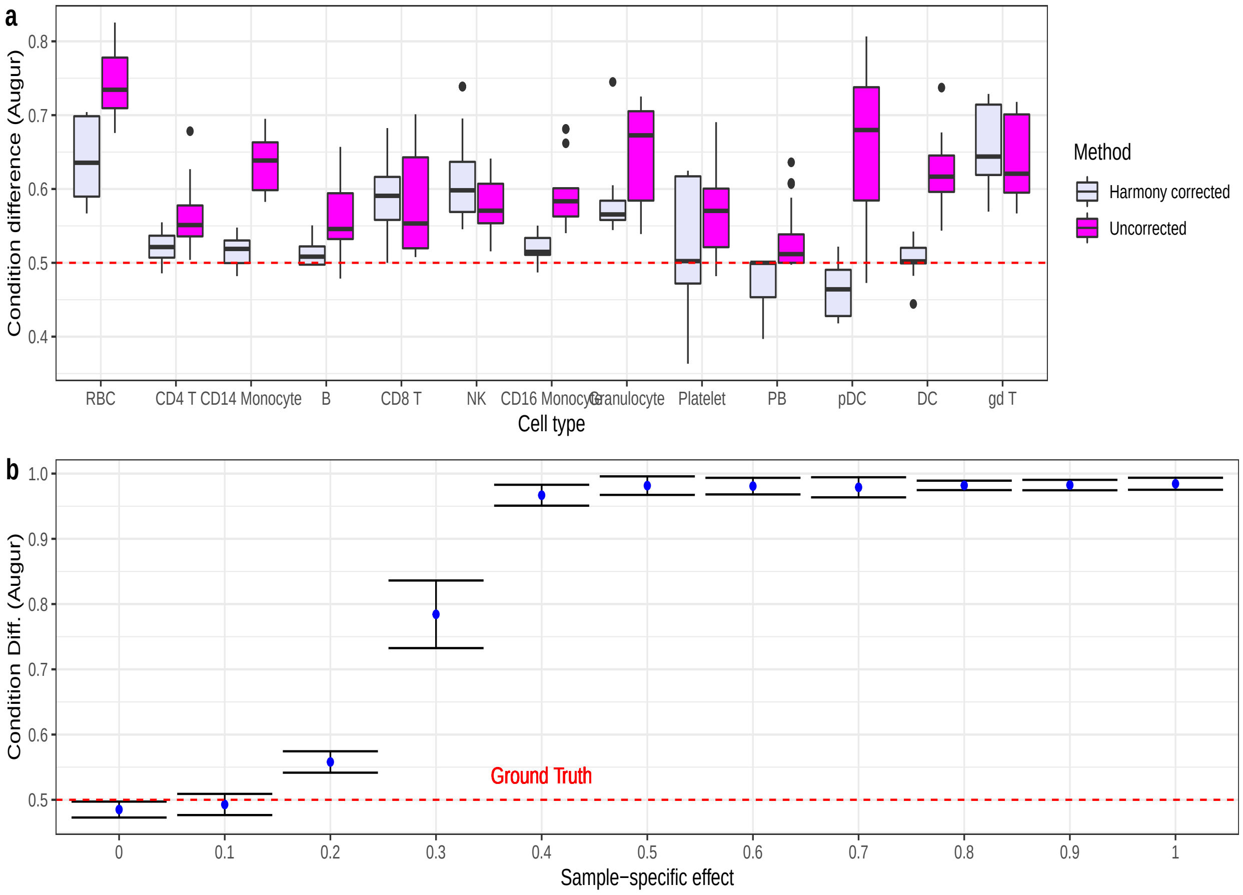

Figure S1: **a.** Repeating the analysis in Figure 1A and using batch correction as a pre-processing step. Each patient was treated as a batch and Harmony (5) was used to obtain batch-corrected PC scores. *Augur* was then applied to the PC scores. **b.** Repeating the same analysis as Figure 1C using the same batch correction procedure mentioned above. Source data are provided as a Source Data file.

D Supplementary Figures

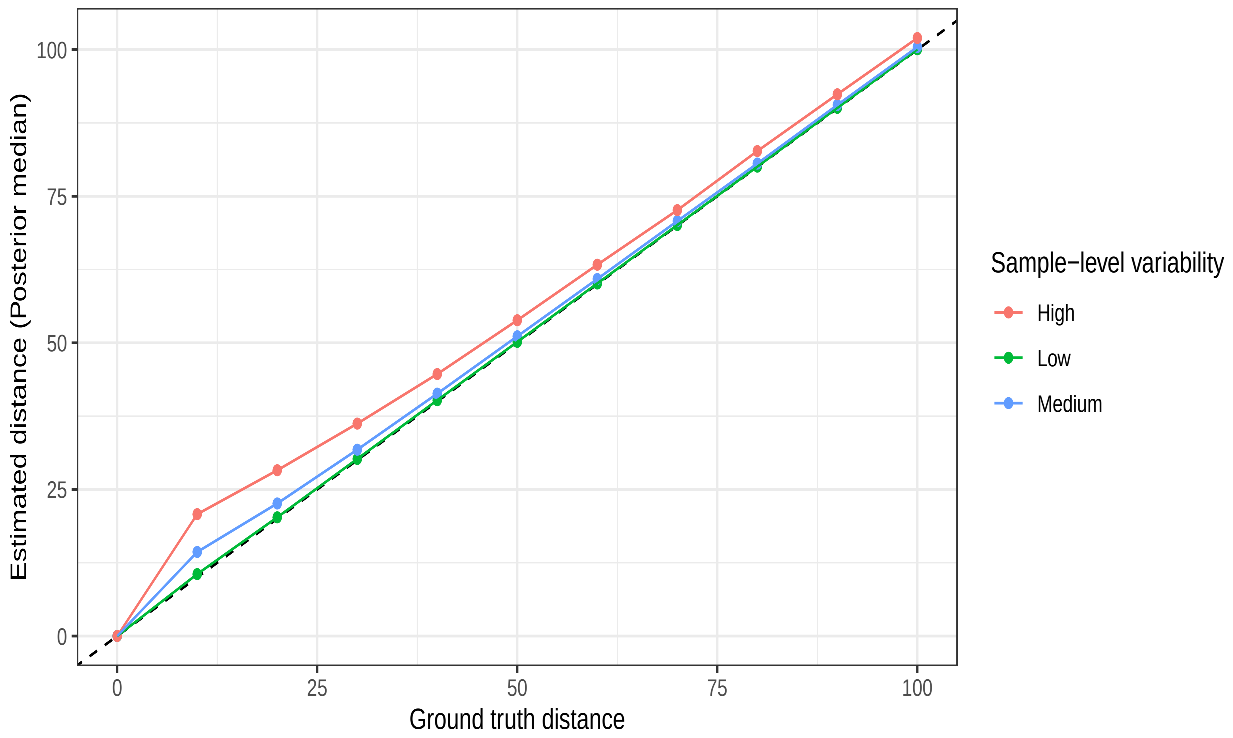

Figure S2: *scDist* recovers ground truth distance on simulated data. For each ground truth distance, the median estimated distance across 100 simulated distances is reported. Data was generated with $G = 1000$, 5 patients per condition, 50 cells per patient. High sample-level variability was defined as $\tau = 1$, medium was defined as $\tau = 0.5$ and low was defined as $\tau = 0.1$. Source data are provided as a Source Data file.

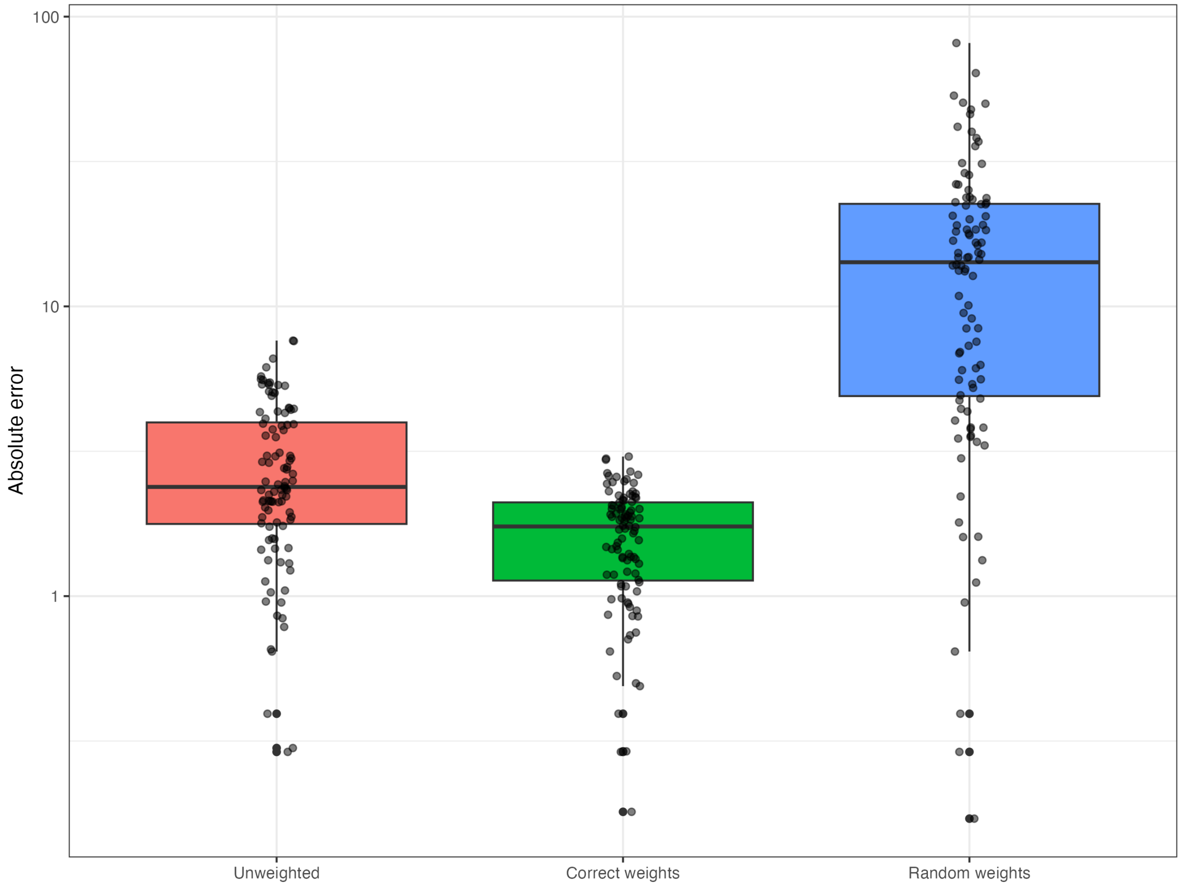

Figure S3: Simulation demonstrating the weighted version of *scDist*. 100 simulated datasets are generated with $\beta_g = 0$ with probability 0.9 and $\beta_g \sim \mathcal{N}(0, 1)$ otherwise. Unweighted sets $w_g = 1$ for all g (this is the default mode of *scDist*). Correct weights sets $w_g = 1$ if $\beta_g \neq 0$ and $w_g = 0$ otherwise. Random weights sets $w_g = 1$ with probability 0.1 randomly. Peformance is assessed by taking the absolute value of the difference between $\sum_g \beta_g^2$ and the estimated distance. Source data are provided as a Source Data file.

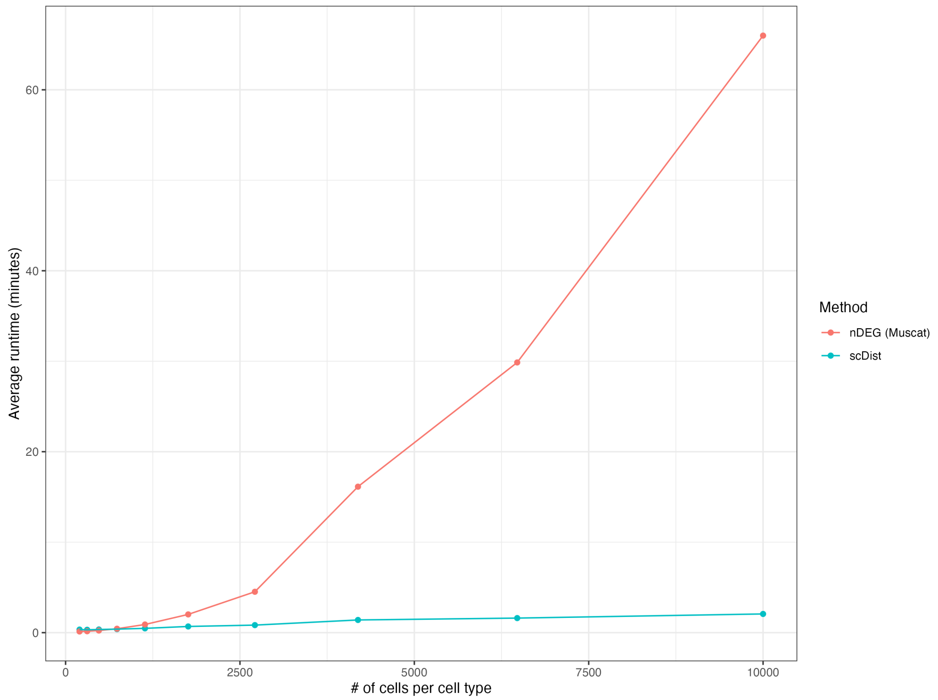

Figure S4: The average runtime (in minutes) of scDist and nDEG (muscat) as the number of cells per cell type varies on the resampled data (see Fig 4a). Source data are provided as a Source Data file.

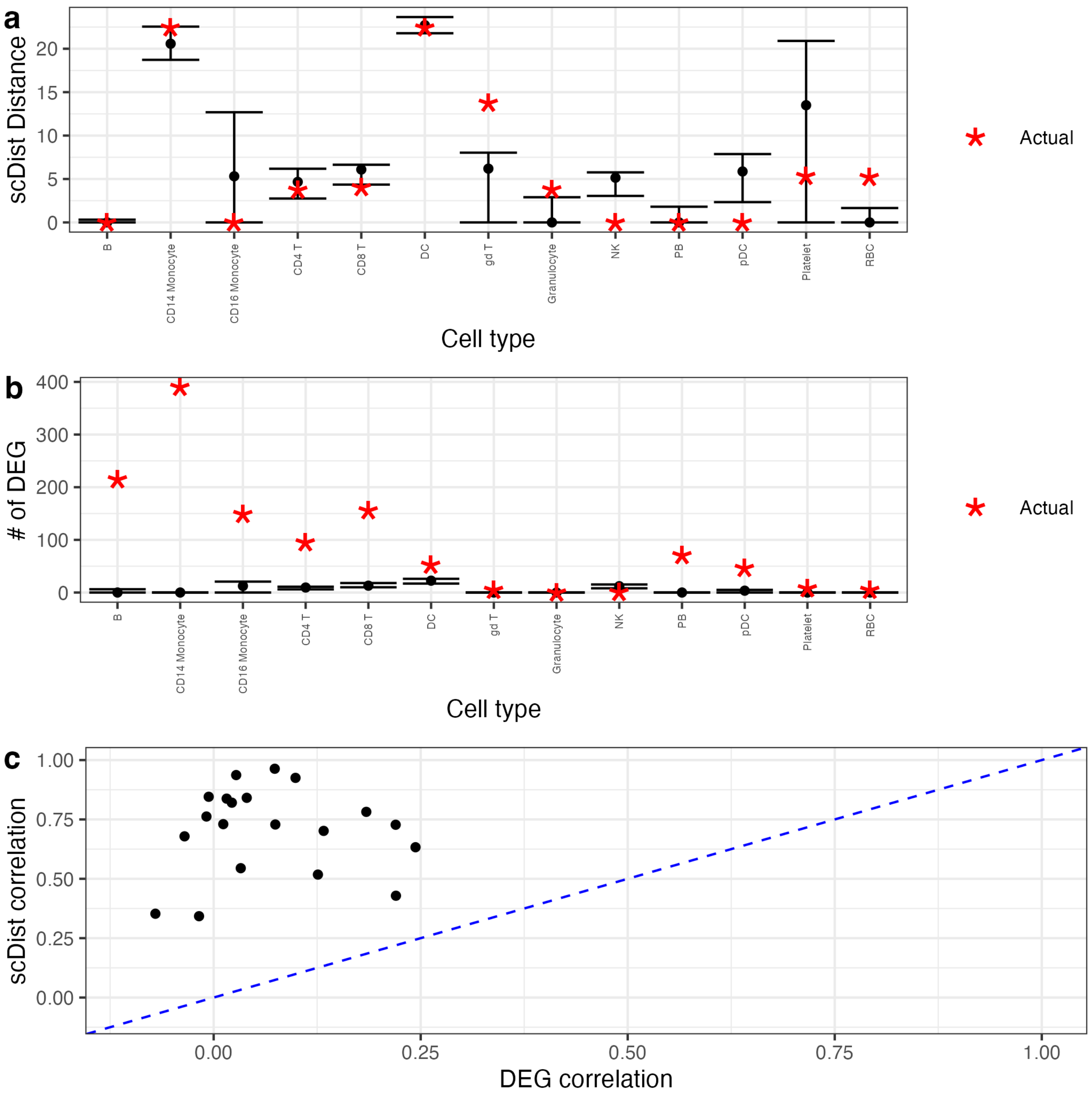

Figure S5: Comparison of *scDist* to counting the number of differentially expressed genes (DEGs) on 20 downsampled datasets. **a.** For scDist, the distance estimates on downsampled data (boxplots) are close to that results from the entire dataset (red star). **b.** The same analysis as in **a** except using the number of DEGs. The pseudobulk differential expression was performed using muscat (6). **c.** For each of the 20 downsampled datasets, the correlation between the downsampled estimates and the estimates on the entire dataset. Values above the dashed identity line show that the correlation is higher for scDist. Source data are provided as a Source Data file.

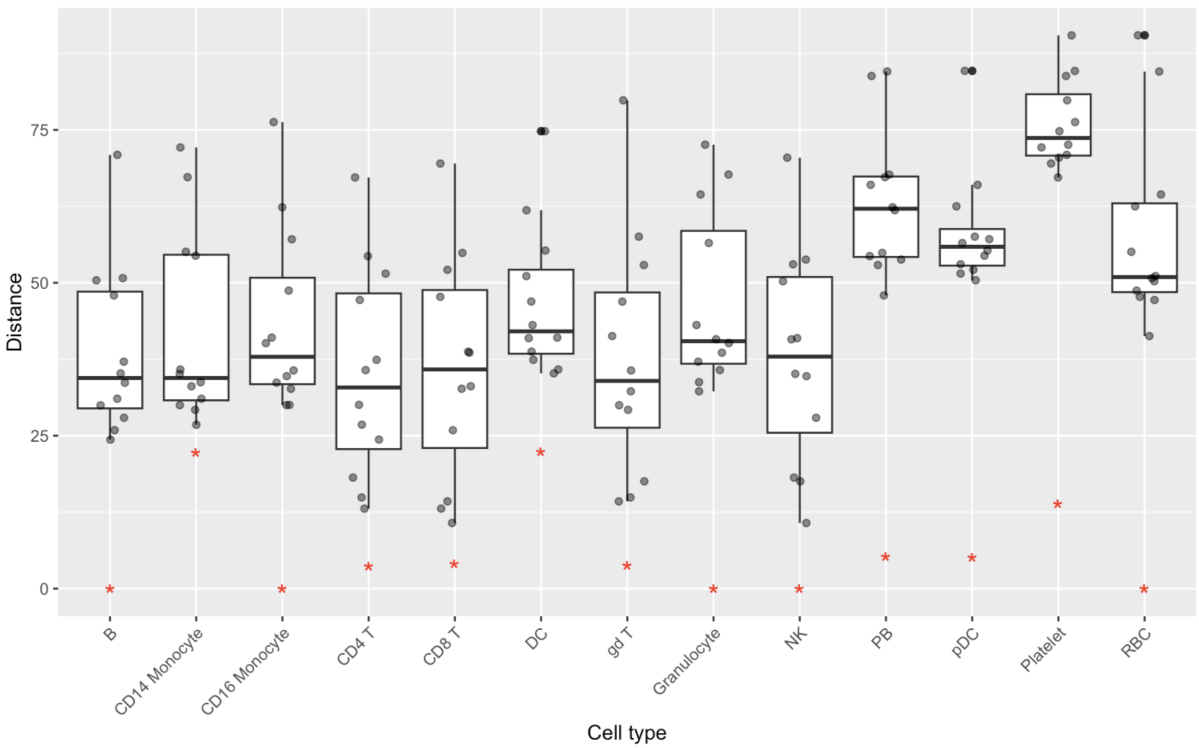

Figure S6: Comparing the inter-condition distance to the inter-cell-type scDist distance estimates on the (7) COVID-19 data. Source data are provided as a Source Data file.

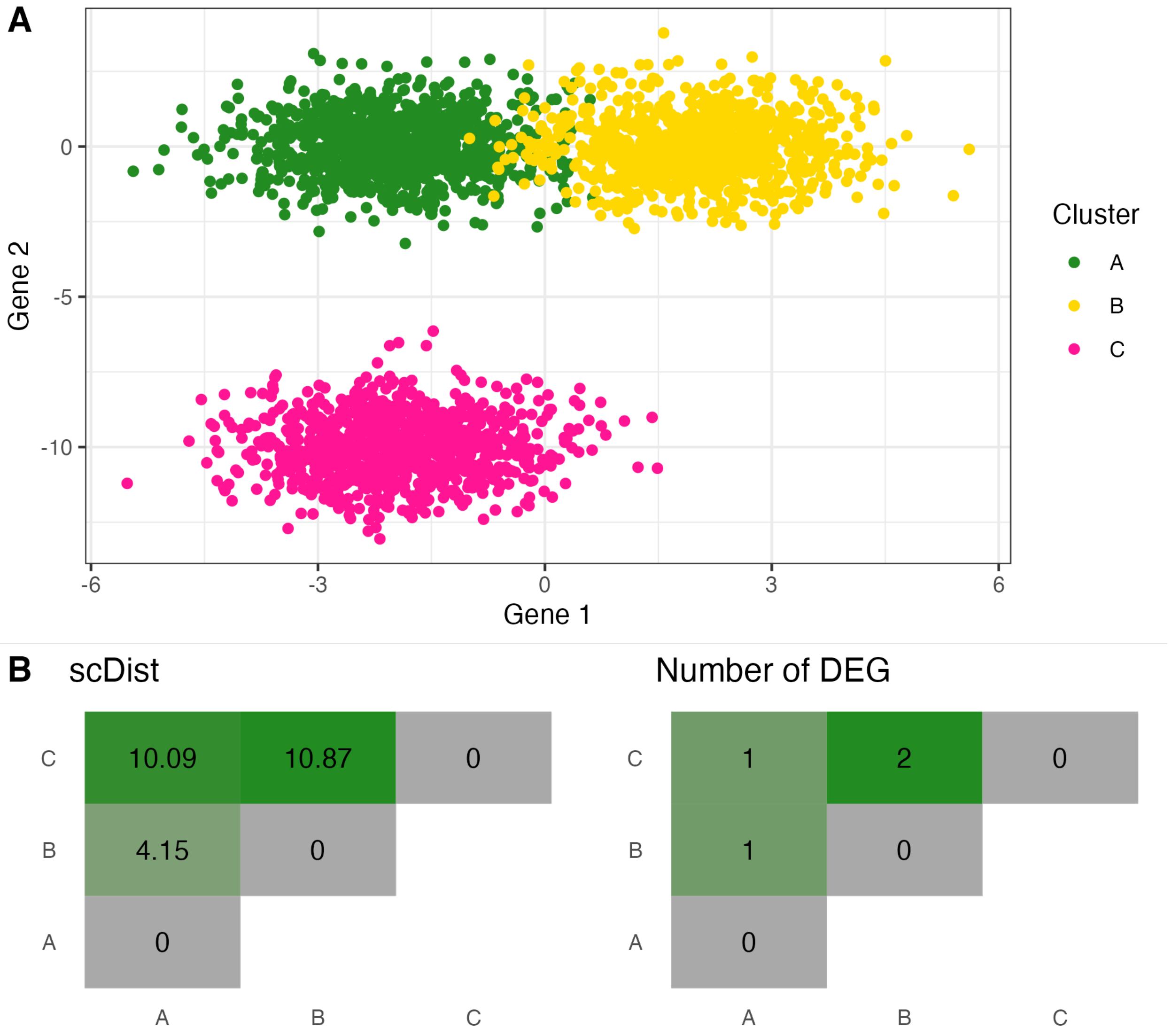

Figure S7: A simulated example showing that counting the number of differentially expressed genes (DEGs) does not account for the magnitude of the expression difference. Three clusters A and B and C with 1000 cells are simulated such that the distance (in gene expression space) between A and B is much smaller than the distance between A and C. However, the number of differentially expressed genes between A and B and A and C is simulated to be 1 for both. **A.** Scatterplot of expression values for the first two genes colored by cluster. The remaining genes (not plotted) are random Gaussian noise (with no cluster differences). **B.** The distance estimates and number of DEGs (computed using a t-test with Bonferroni adjust p-value cutoff of $0.05/10^3$) for all pairwise comparisons.

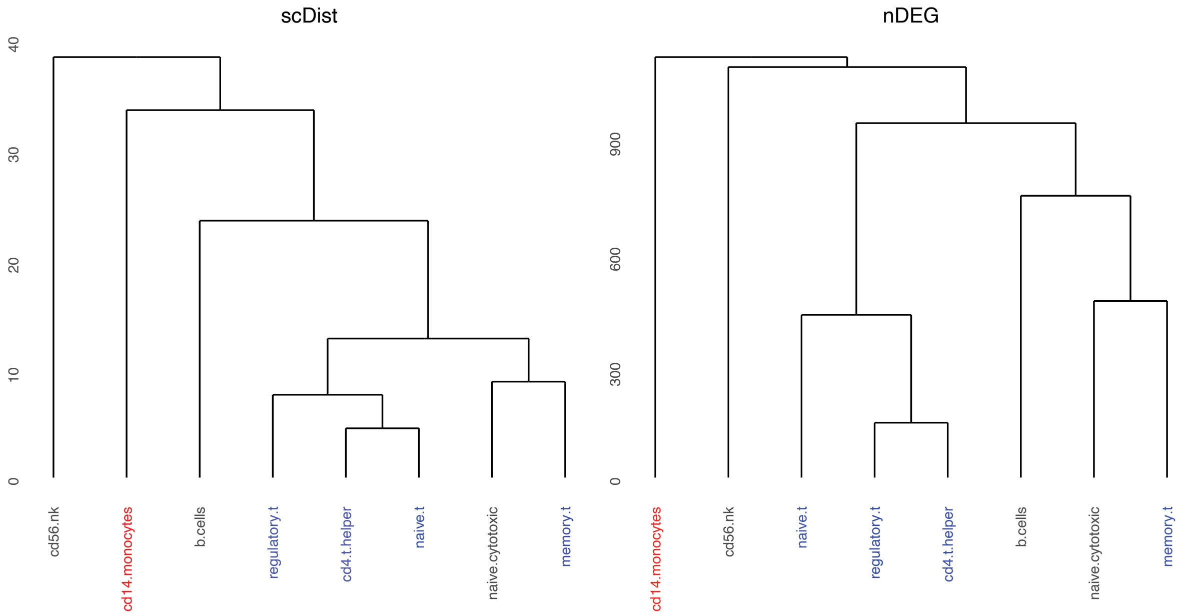

Figure S8: Repeating the analysis of Figure 4c,d without subsampling the CD14 Monocytes to have a smaller sample size. Source data are provided as a Source Data file.

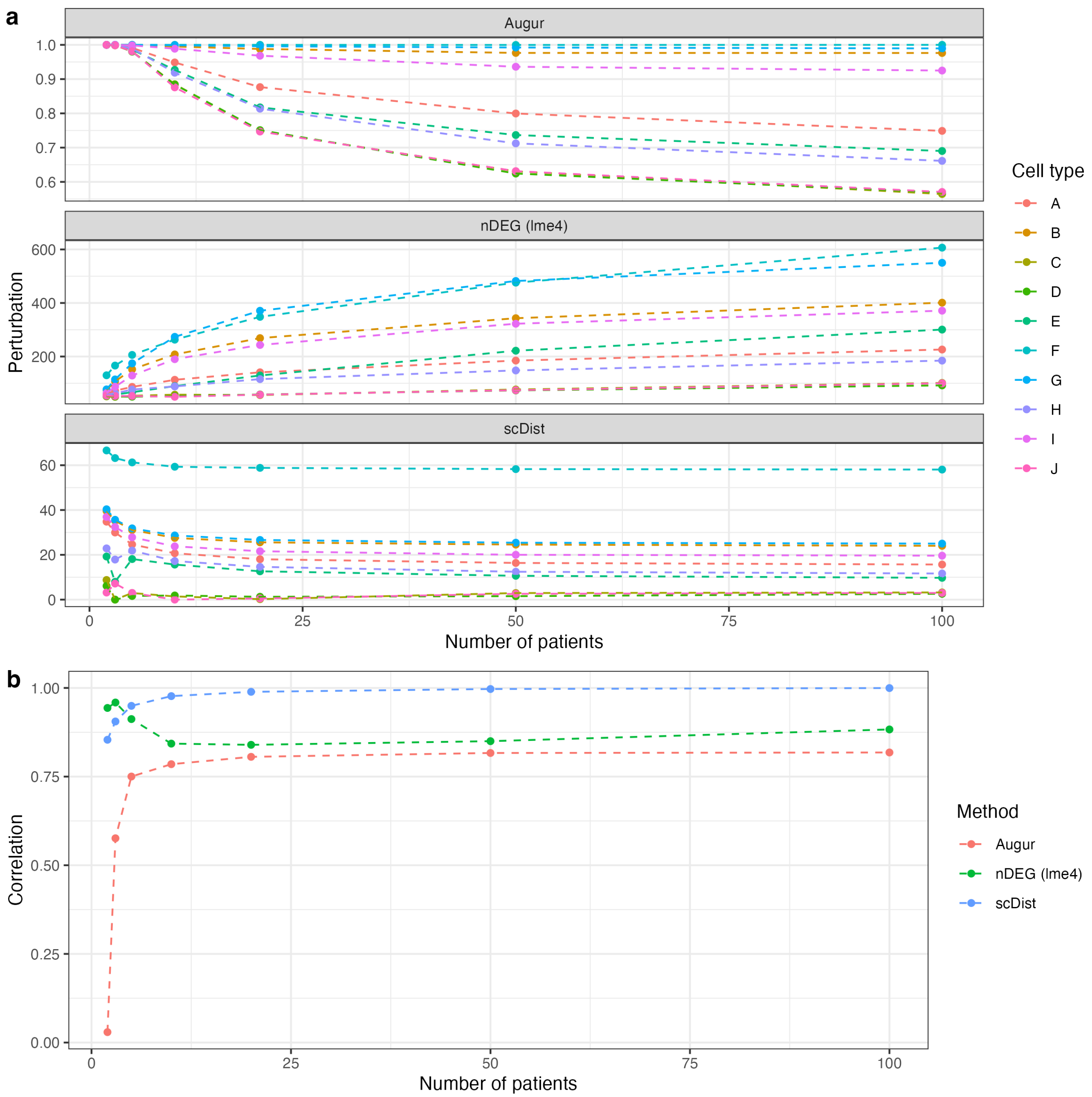

Figure S9: **a** Comparison of scDist, Augur, and nDEG (this time computed using lme4 (8)) on simulated data as the number of patients increases. **b** The correlation between the ground truth perturbation (distance) as the number of patients increases. Source data are provided as a Source Data file.

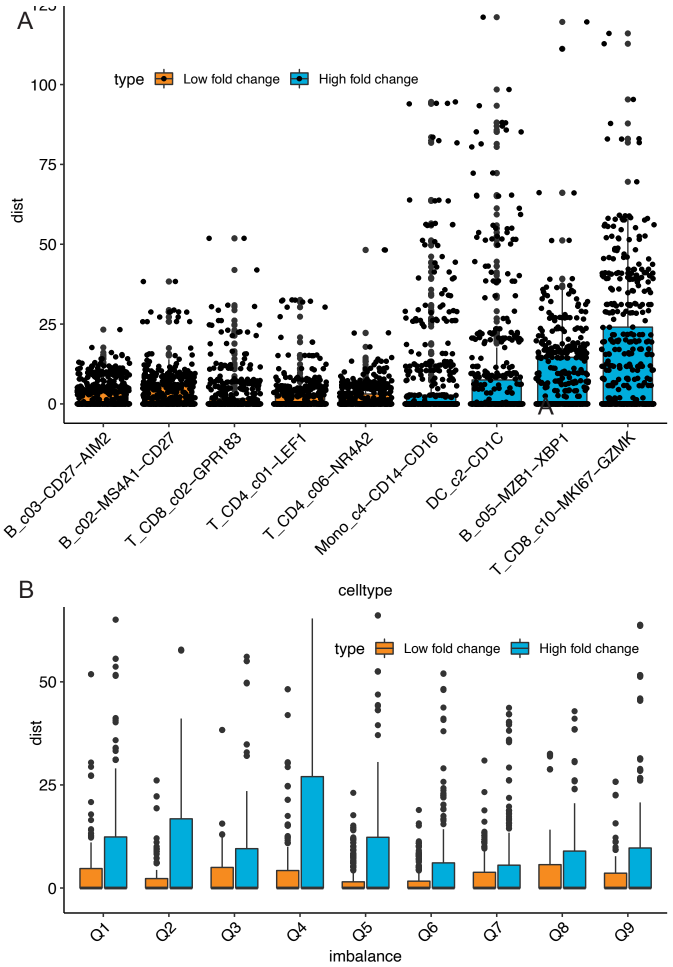

Figure S10: **A.** Median *scDist* distances with Bayesian correction, depicting probable true (blue) and false (orange) positive cell types. **B.** Median distances estimated by *scDist* illustrating the influence of cell number variation in subsampled datasets (of false positive cell types).

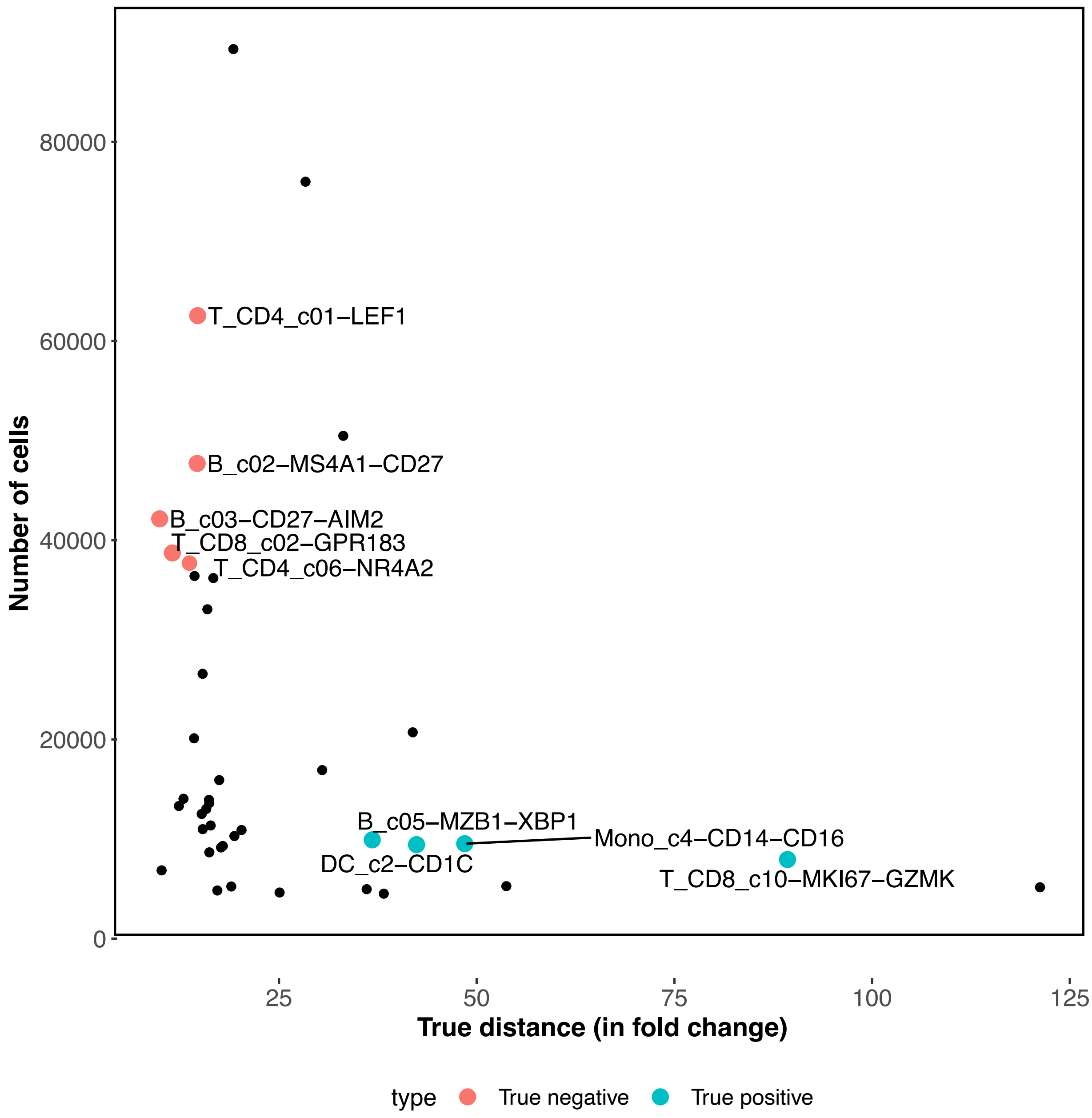

Figure S11: Ground truth positive (True positive) and negative (True negative) sets for Ren. et. al. COVID-19 Single-Cell Cohort (284 Samples). The plot displays the average gene fold changes between COVID-19 and control samples for various cell types, with the number of cells in the cohort on the y-axis. Highlighed are cell types chosen true positive and true negative.

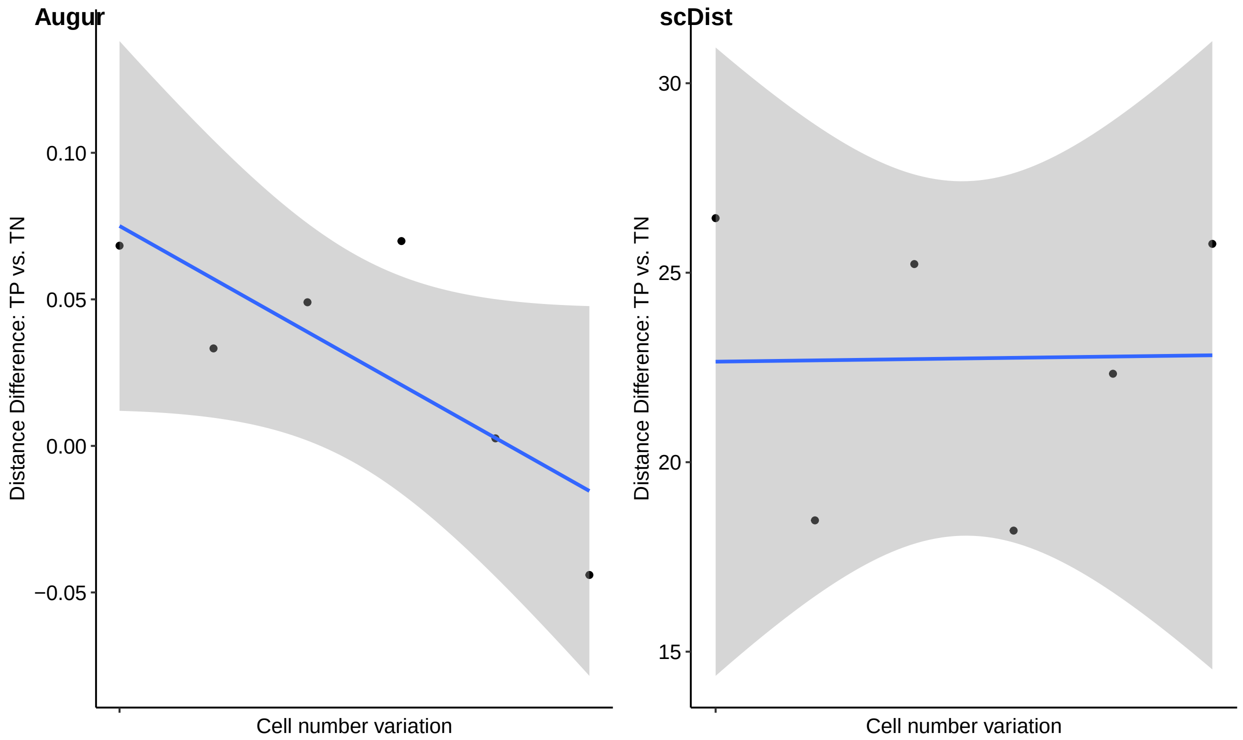

Figure S12: Difference of distances for ground truth positive (TP) and negative (TN) against the cell number variation for Augur (left panel), and scDist (right panel).

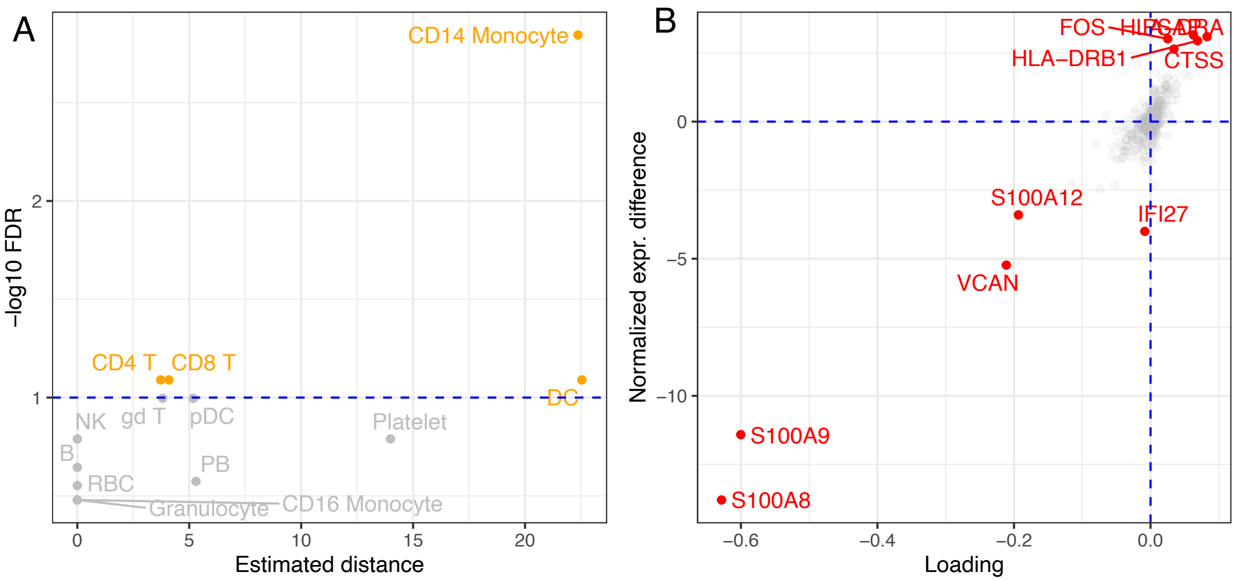

Figure S13: **A.** Estimated difference for each cell type in the COVID-19 data is plotted against the false discovery rate (FDR). **B.** Focusing on CD14+ monocytes, the PC1 weight for each gene is plotted against its expression difference (comparing cases and controls); red-colored genes have the highest contribution to the identified perturbation in CD14+ monocytes.

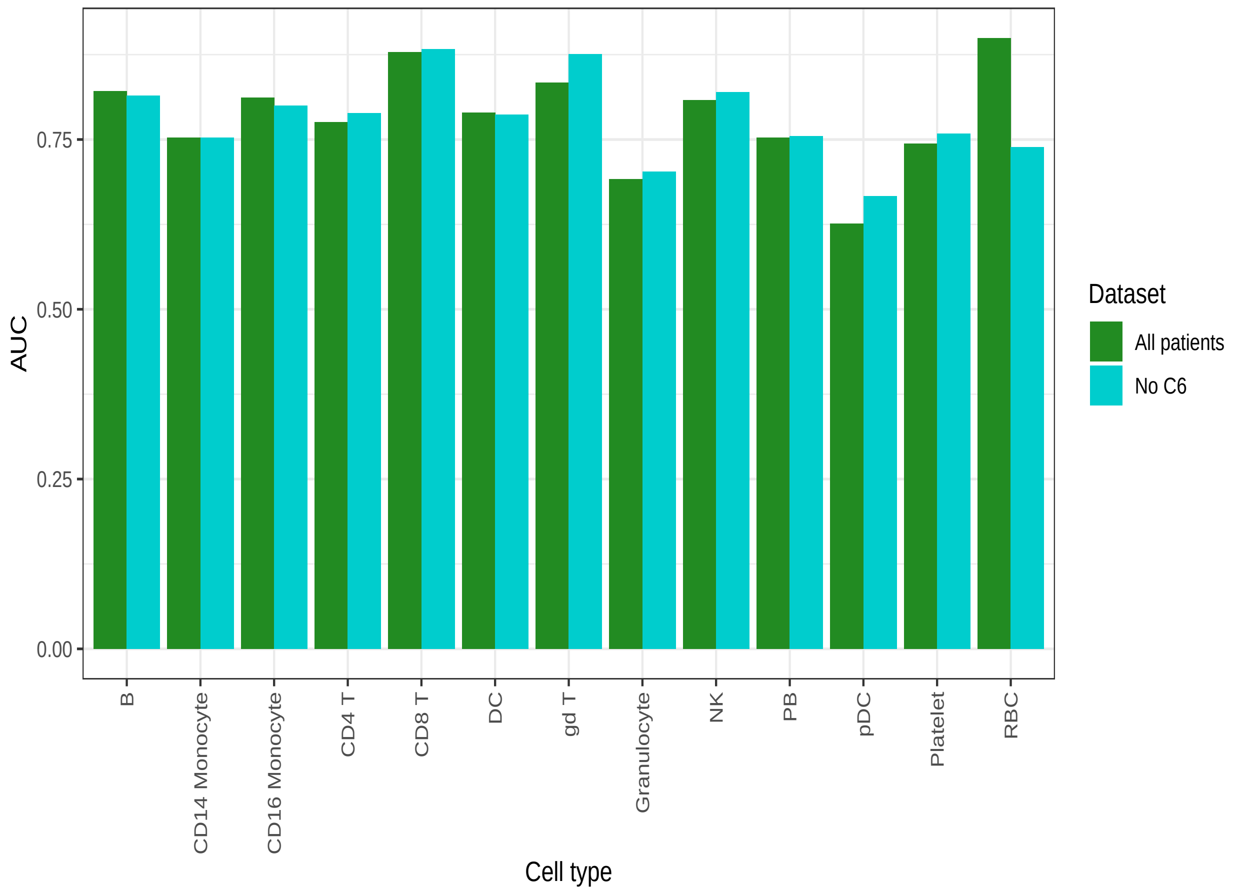

Figure S14: Applying *Augur* to the (7) data with the sixth case (patient C6) removed.

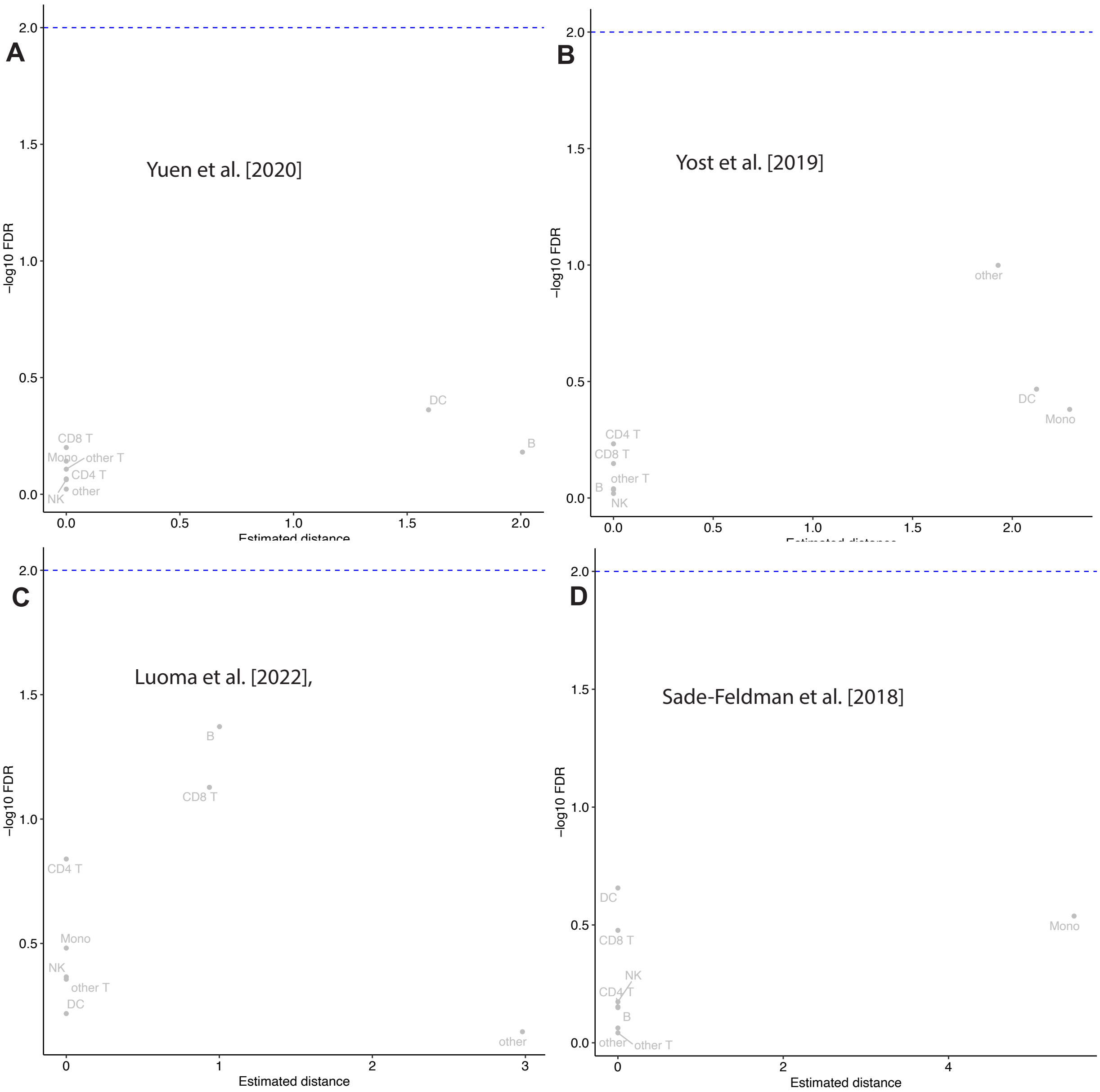

Figure S15: **Analysis of Responder and Non-responder Groups Using *scDist* in Four Single-Cell Cohorts.** Application of *scDist* to compare responders and non-responders in four independent single-cell cohorts. No significant cell types were identified in any of the cohorts. Panels A-D are based on data from Yuen et al. (9) (**A**), Yost et al. (10)(**B**), Luoma et al. (11) (**C**), and Sade-Feldman et al. (12) (**D**).

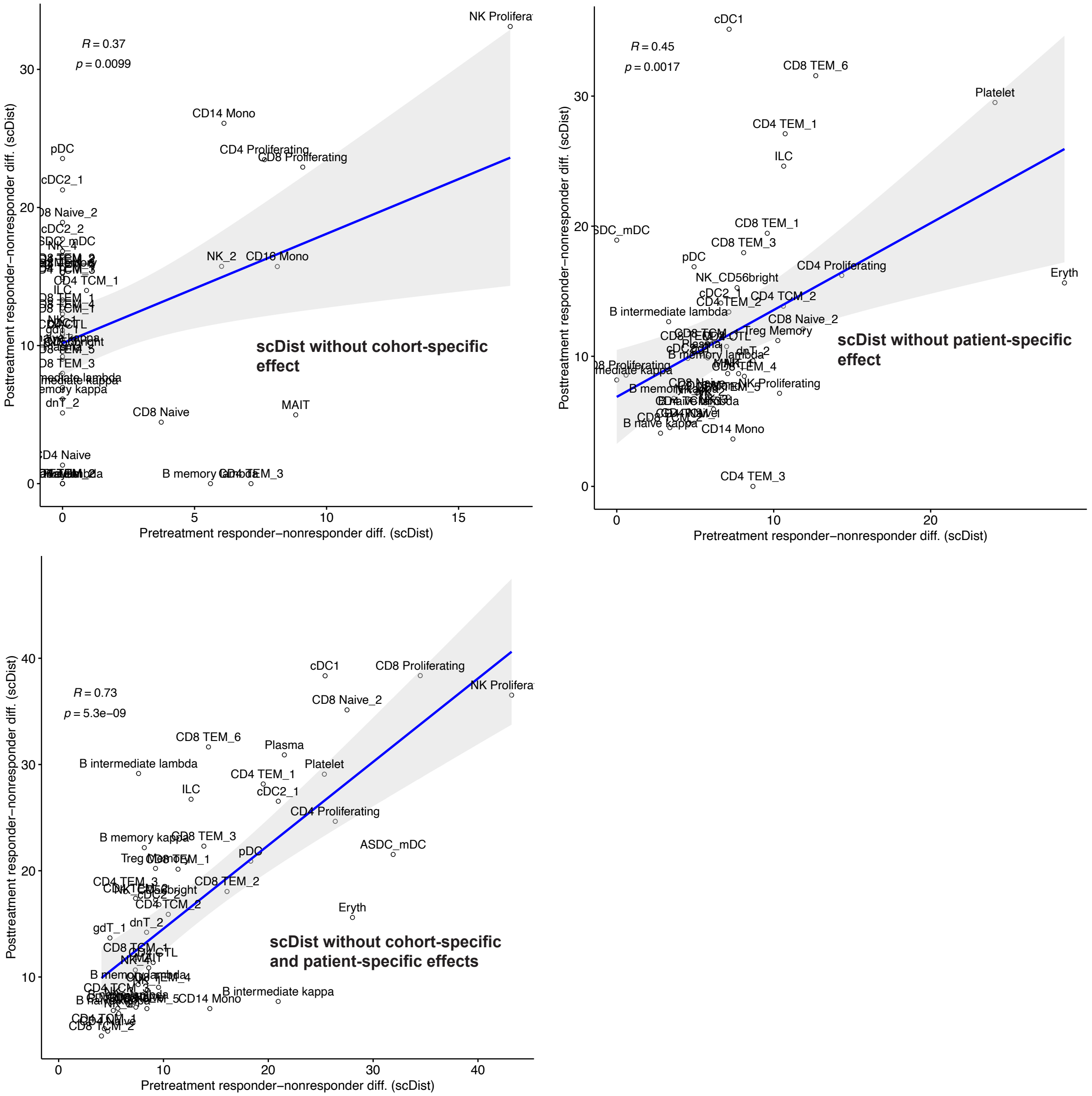

Figure S16: **Evaluating Performance of *scDist* with and without confounding effects.** Pretreatment and posttreatment sample differences were estimated using *scDist* without A) cohort-specific effects, B) patient-specific effects, and C) cohort and patient-specific effects.

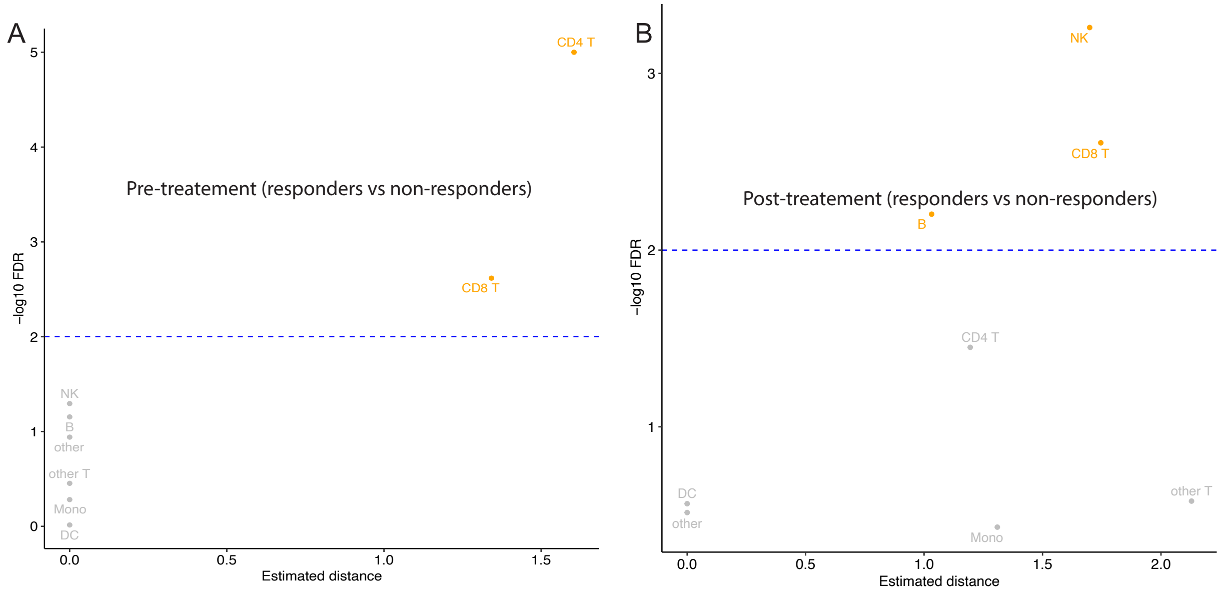

Figure S17: **Comparing Responders and Nonresponders Using *scDist* in Integrated Single-Cell Cohorts.** Application of *scDist* to assess differences between responders and non-responders in an integrated analysis of single-cell cohorts. **A)** Pretreatment differences and their significance. **B)** Posttreatment differences and their significance.

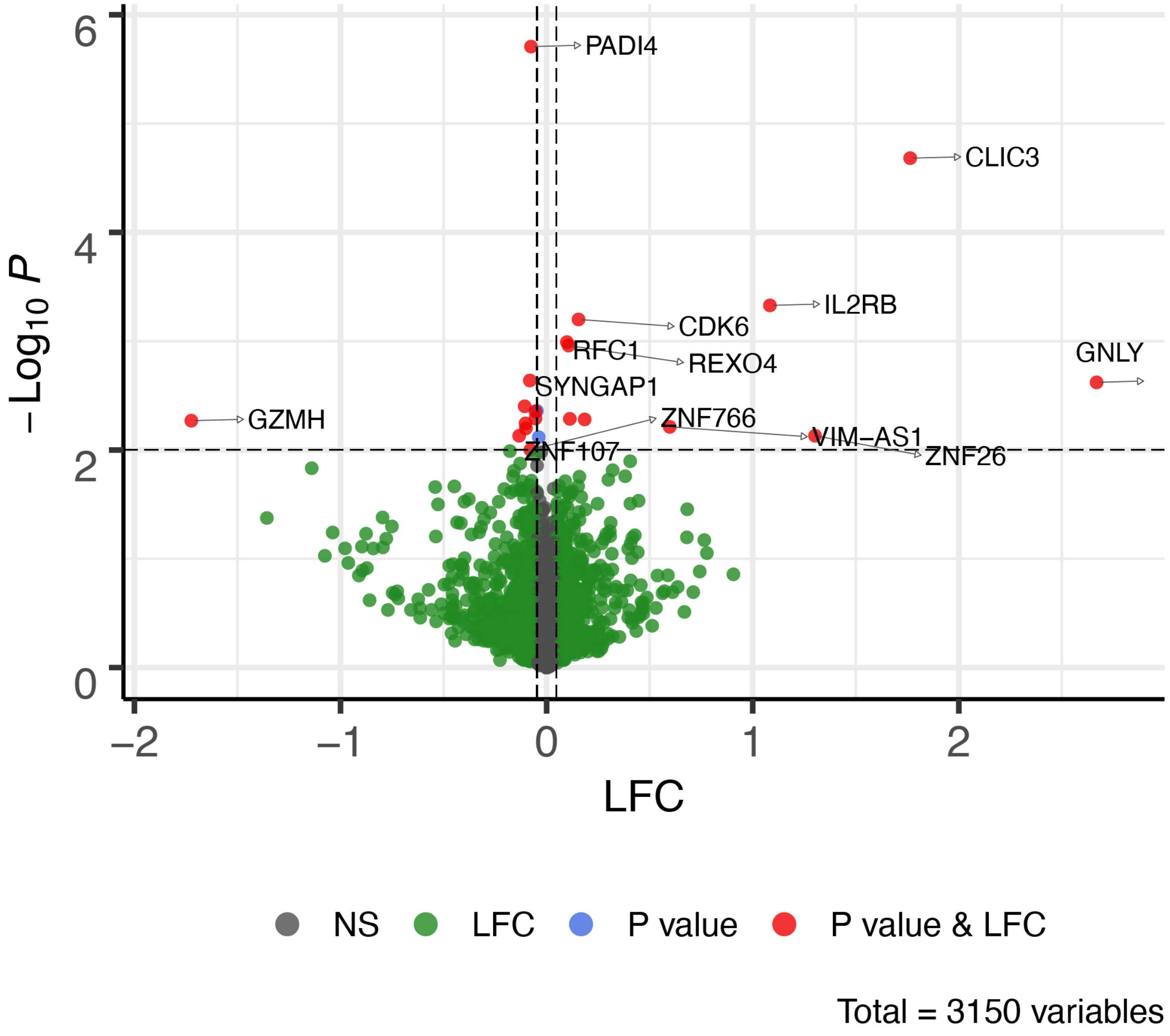

Figure S18: **Differential expression analysis of responders and non-responders in NK-2 cells from integrated single-cell cohorts.** A volcano plot illustrating the differential expression between responders and non-responders within NK-2 cells from integrated single-cell cohorts. The plot displays both the magnitude of differential expression and its statistical significance.

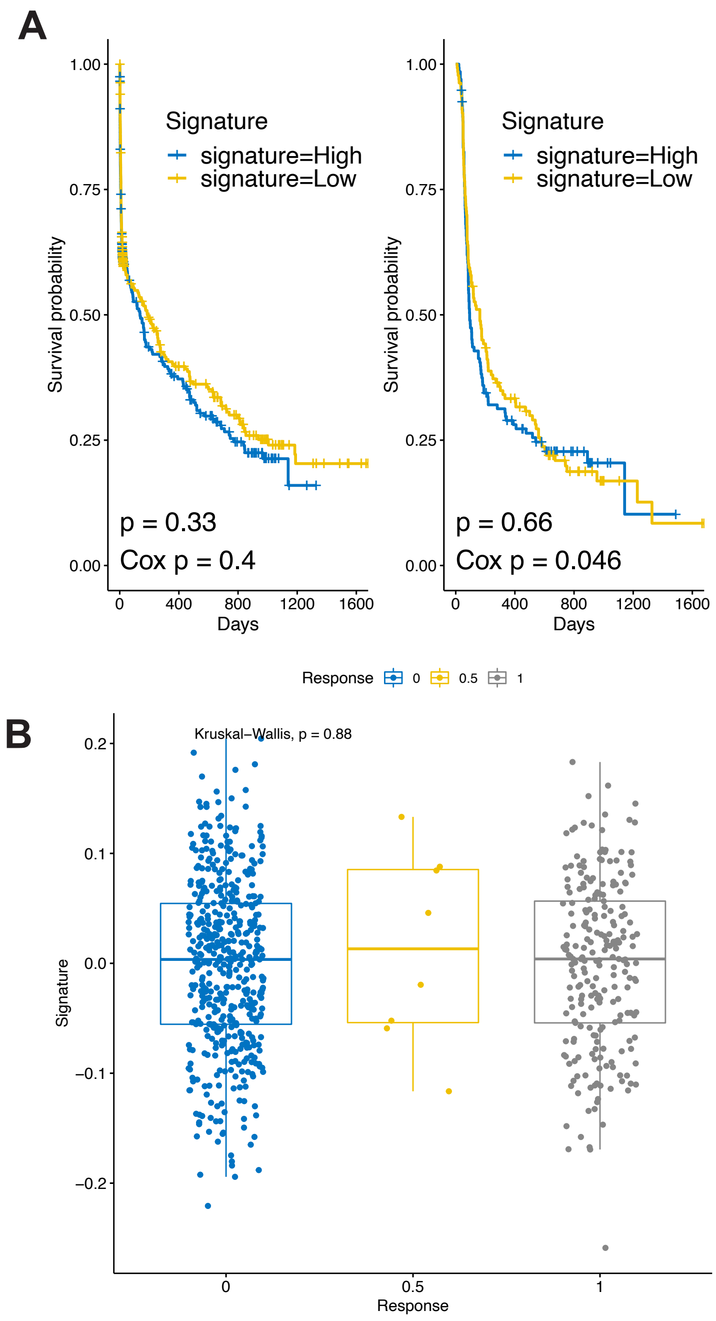

Figure S19: A. Kaplan-Meier plots display the survival differences in anti-PD-1 therapy patients, categorized by low-risk and high-risk groups using the median value of the Plasma signature value; overall and progression-free survival is shown. **B)** Plasma signature levels in non-responders, partial-responders, and responders (Bulk RNA-seq cohorts).

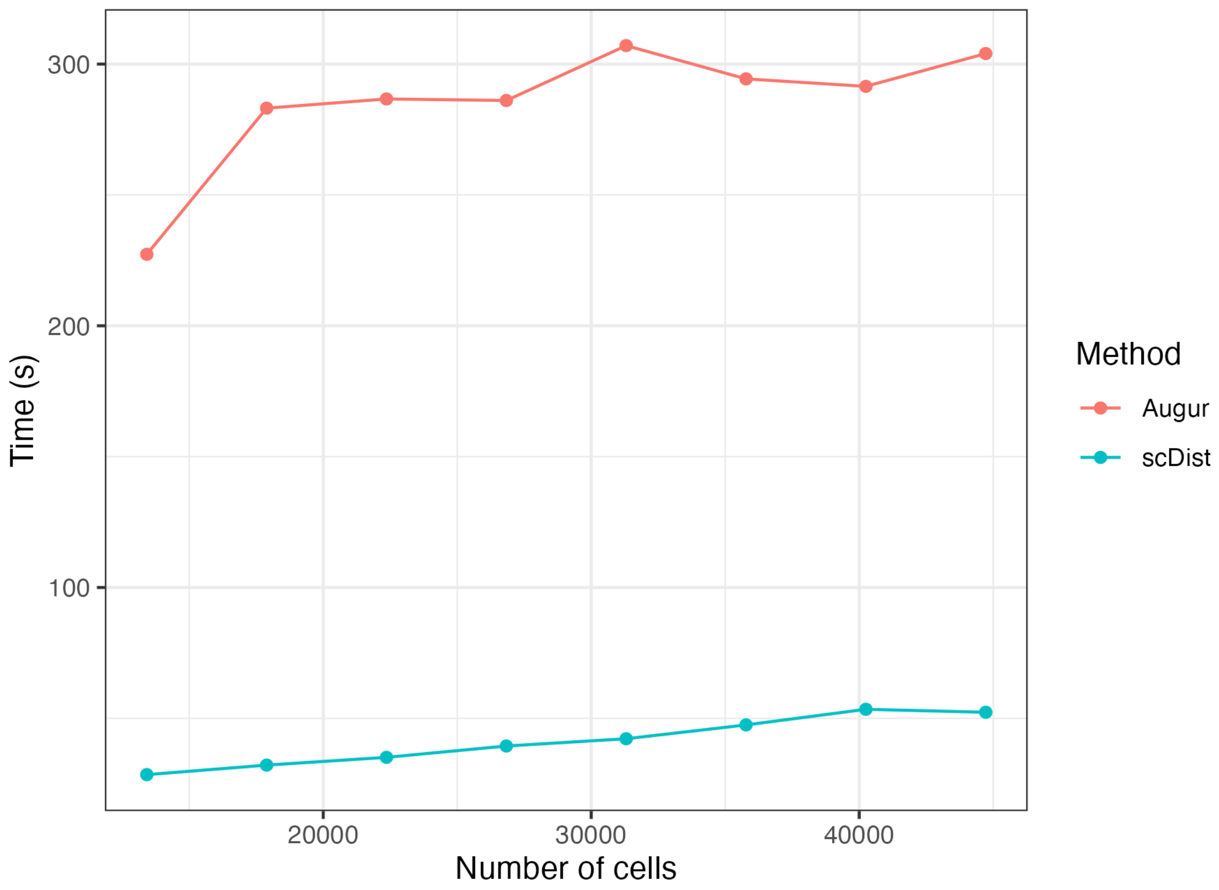

Figure S20: Comparison of the run-time between *Augur* and *scDist* on random subsets of the (7) data.

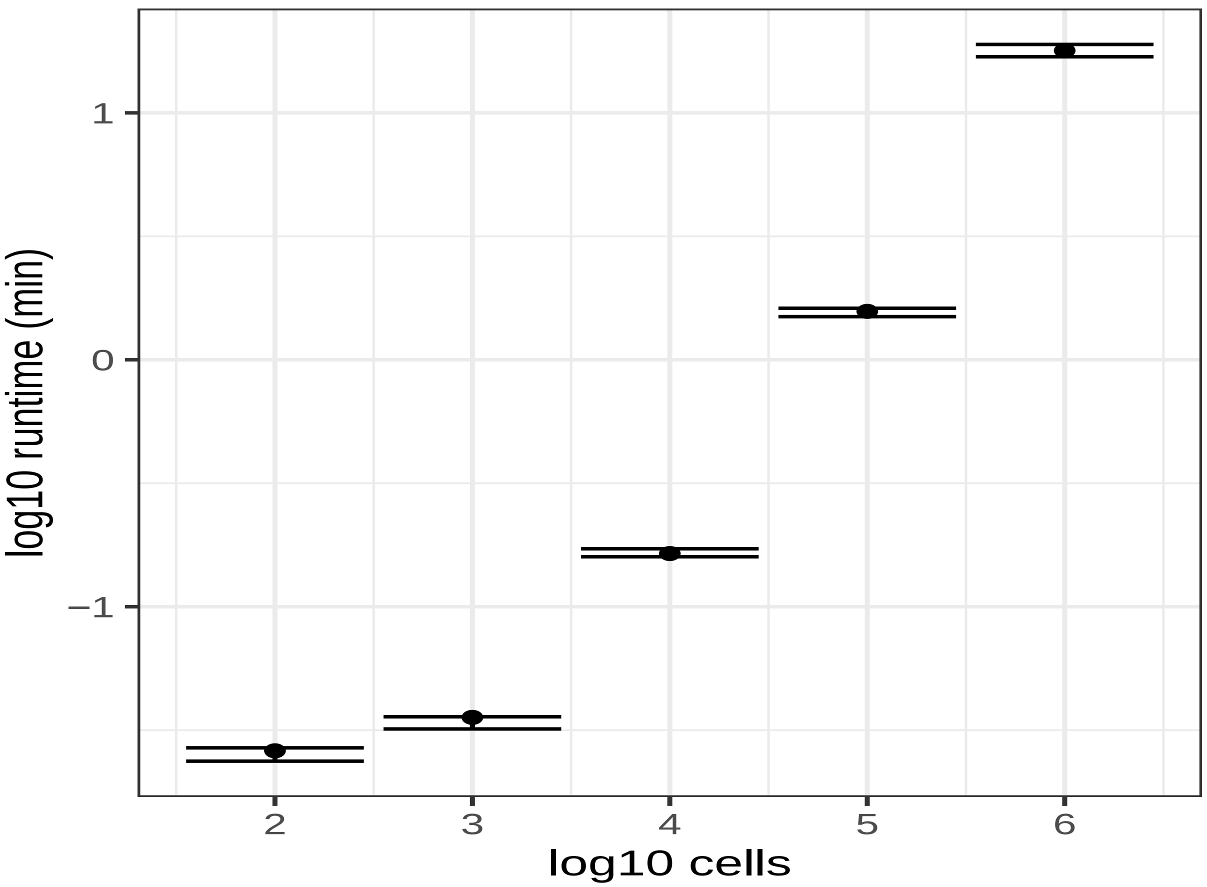

Figure S21: Log-log plot of runtime for *scDist* for simulated datasets with varying number of cells. Median and interquartile range across 10 iterations are reported for each size. 10 patients were simulated with an equal number of cells per patient. Data was generated with $G = 1000$, 5 patients per condition, 50 cells per patient, and a patient level random effect $\tau^2 = 1$. Source data are provided as a Source Data file.

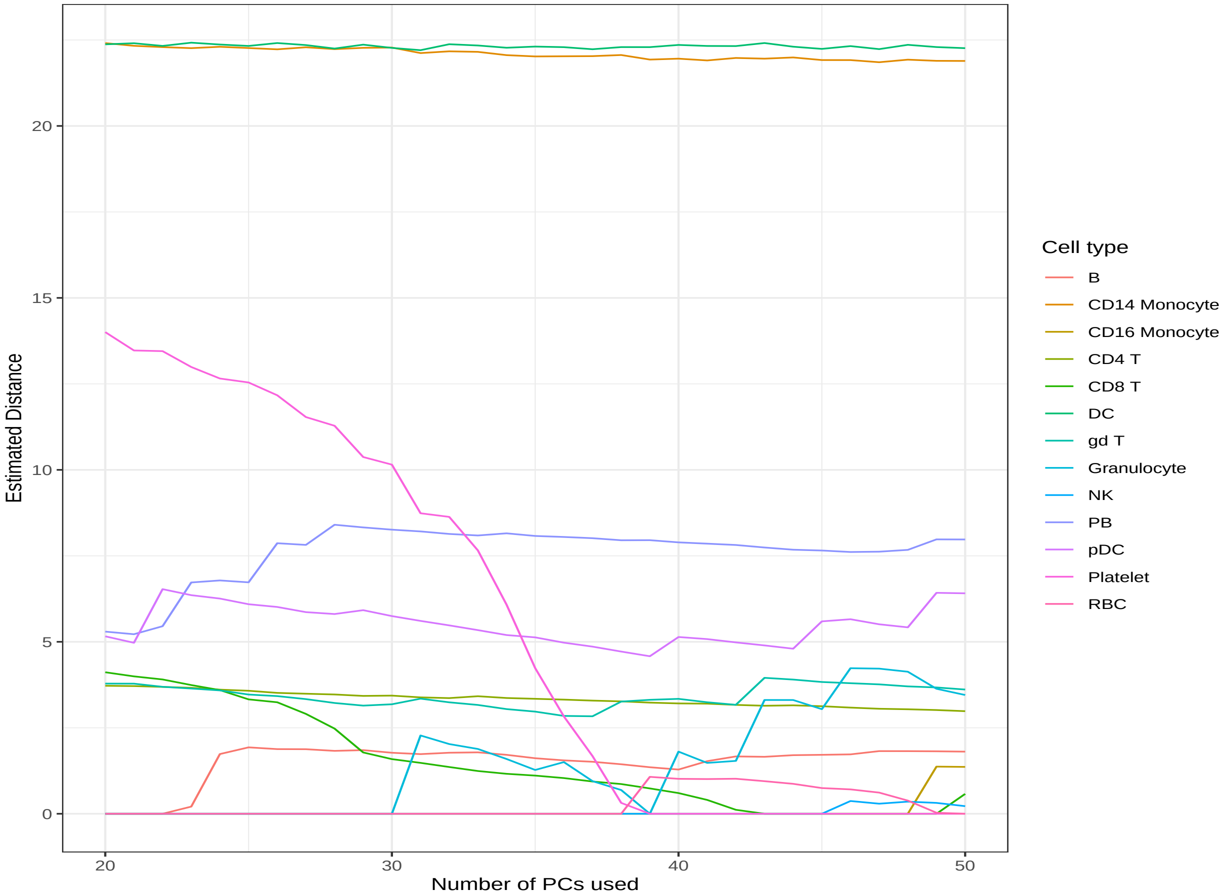

Figure S22: On the (7) dataset, distances were estimated using $20 \leq K \leq 50$ PCs. For a majority of the cell types, the estimated distance is stable as K varies. Source data are provided as a Source Data file.

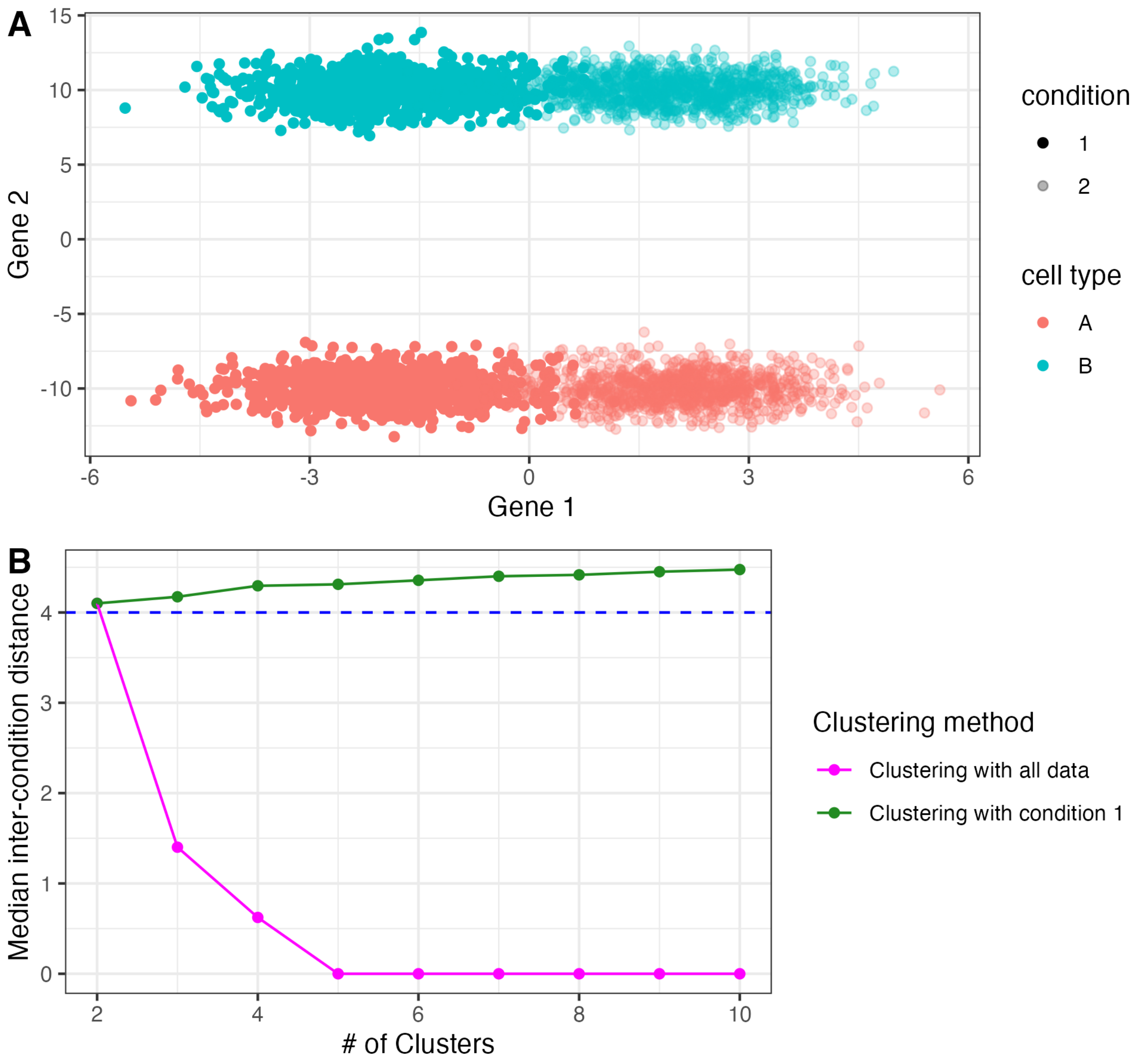

Figure S23: **A.** Simulated example with two true cell types, each with a condition distance of 4. The condition distance is entirely contained within gene 1 and the cell type distance is entirely contained within gene 2. The remaining 998 genes were simulated to be Gaussian noise. **B.** k-means with various choices of k was used to cluster the data and the median condition-distance between all clusters was measured. In the first approach, all the data is used for clustering. In the second approach, only condition 1 is used for clustering, and then all cells in condition 2 are assigned a label by finding the nearest centroid from the previous step.

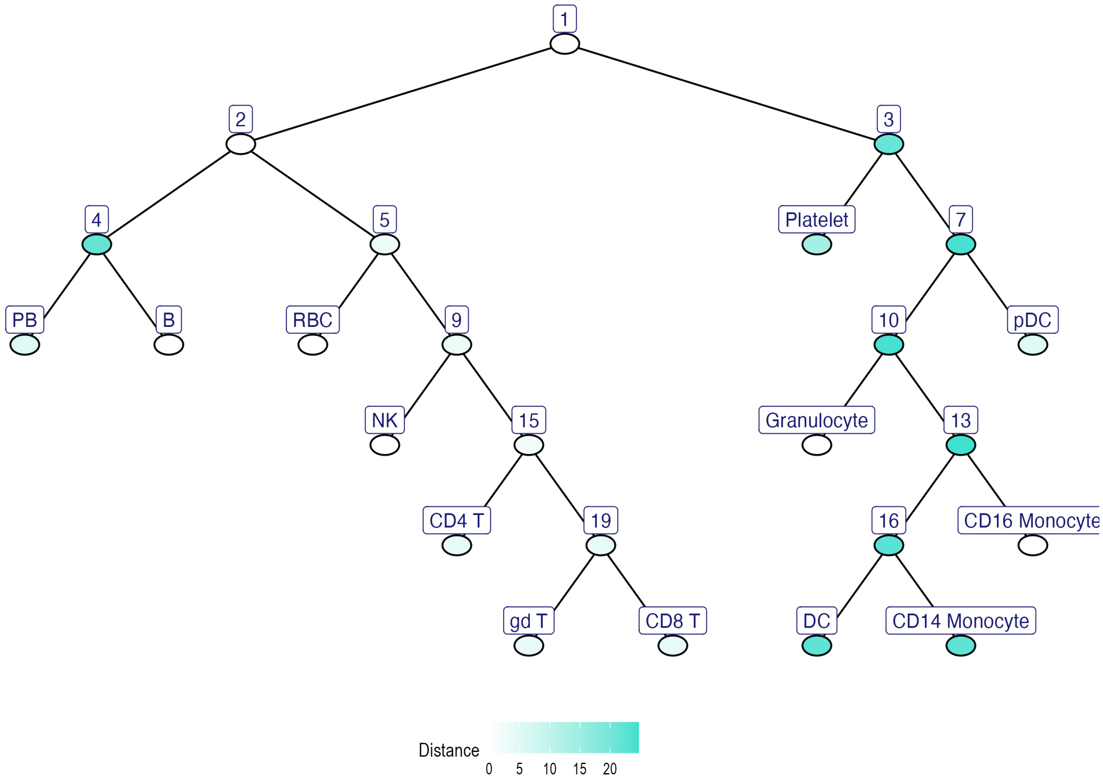

Figure S24: On the COVID-19 data of (7), a cluster tree is estimated (Appendix) that summarizes the relationships between cell types. Then scDist is applied at each internal node of the tree, where all cell types that descend from that node are combined into a single cluster. This tree-based approach can be used to diagnosis problems associated with over-clustering.

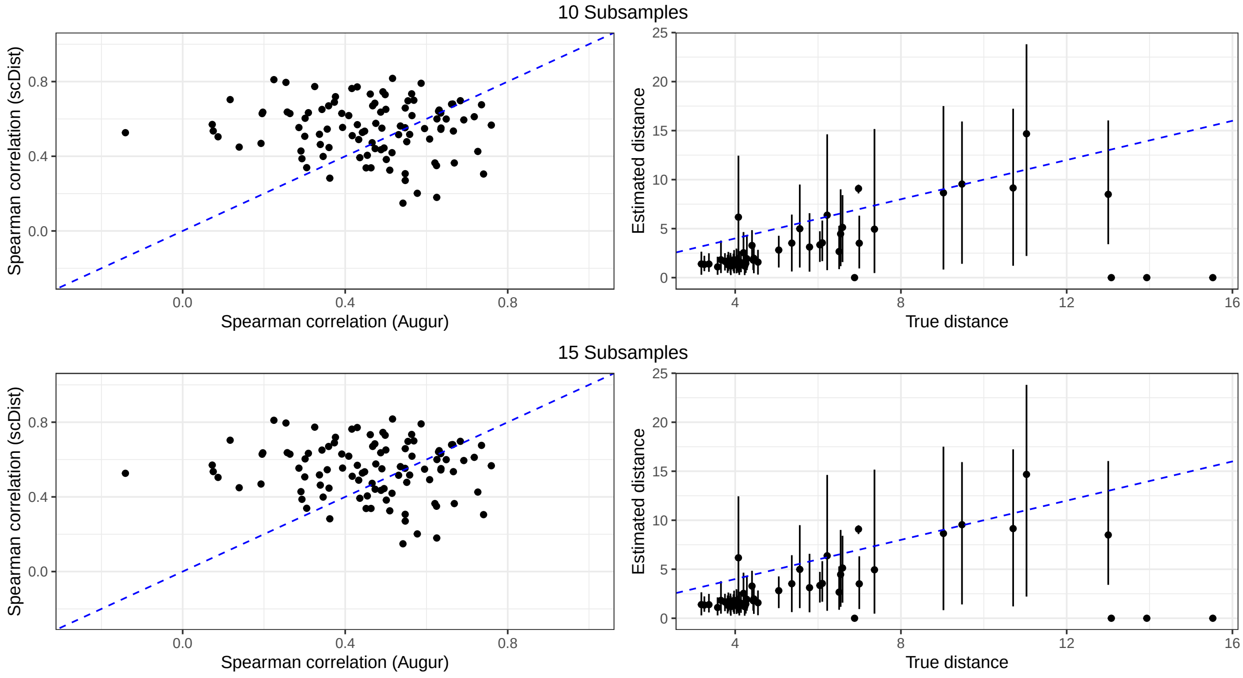

Figure S25: Repeating the subsampling analysis with more patients. Top: Figure 5a and Figure 5b used 5 samples per condition. Bottom: Repeating the analysis with 10 subsamples per patient. With 15 subsamples, the average spearman correlation is higher (0.44 vs 0.54), the mean width of the error bars is lower (6.01 vs 4.68), and the correlation with the ground truth distance is slightly higher (0.30 vs 0.39). Source data are provided as a Source Data file.

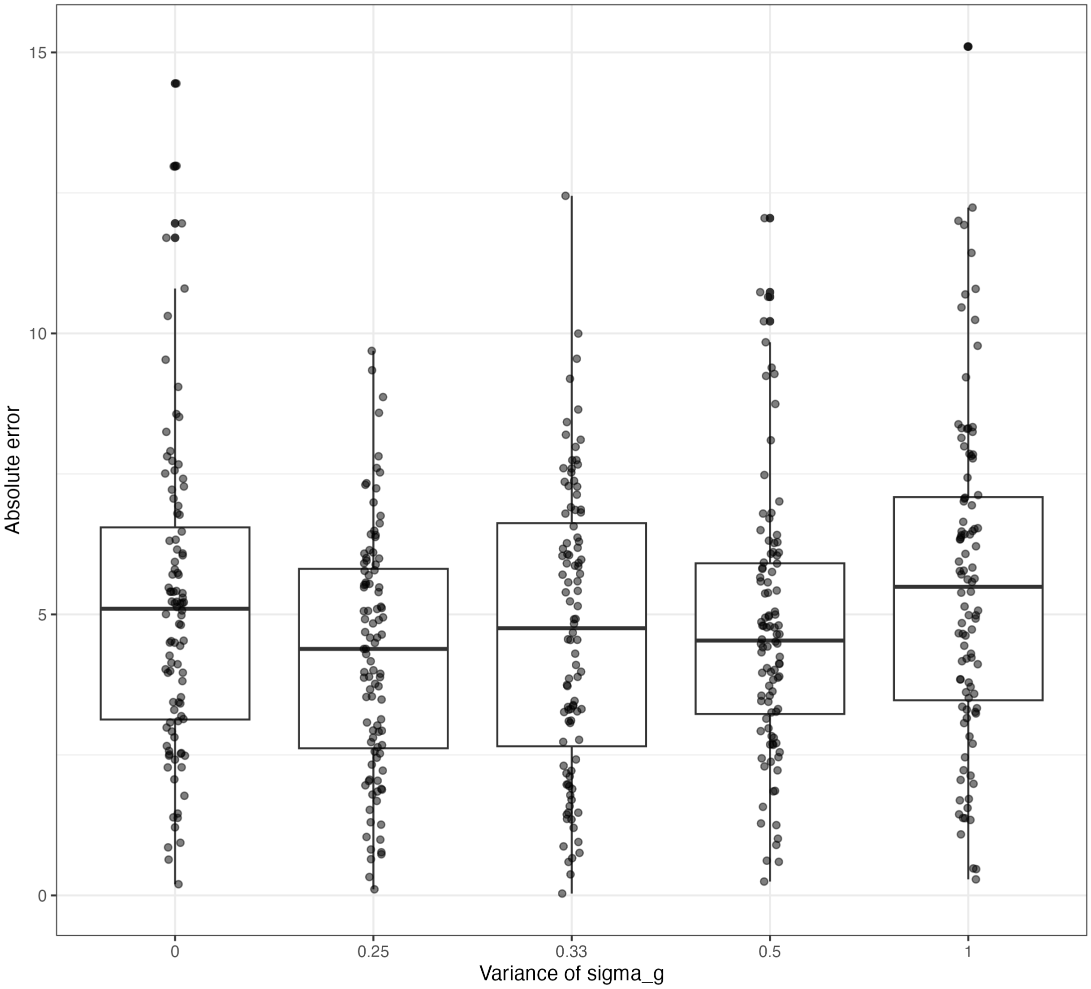

Figure S26: Testing robustness of *scDist* to the assumption that τ and σ are shared across genes. We simulated datasets where each gene has an individual (random) σ_g and τ_g. Specifically, we drew $\sigma_g \sim \text{Gamma}(r, r)$ and $\tau_g \sim \text{Gamma}(r/2, r)$ so that $\text{Var}(\sigma_g) = 1/r$. As r varies, the estimation accuracy of the distance D does not change significantly. Source data are provided as a Source Data file.

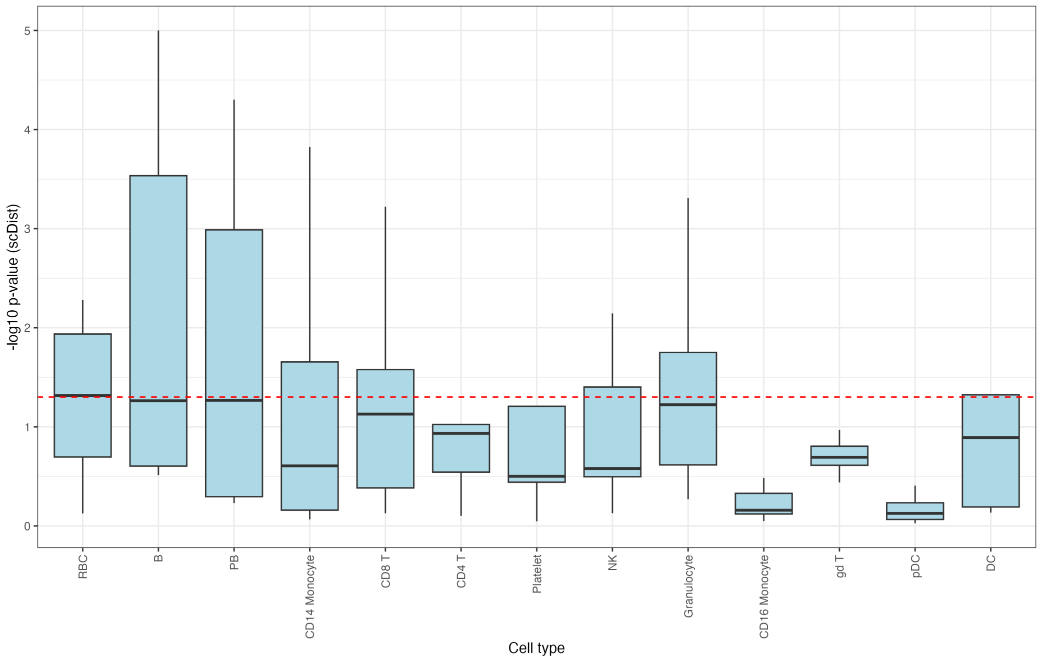

Figure S27: Repeating the analysis of Figure 3b using the FMT method (2) (Appendix Comparison to p-values from FMT) of lmerTest. The dashed red line indicates the $p = 0.05$ cutoff. Source data are provided as a Source Data file.

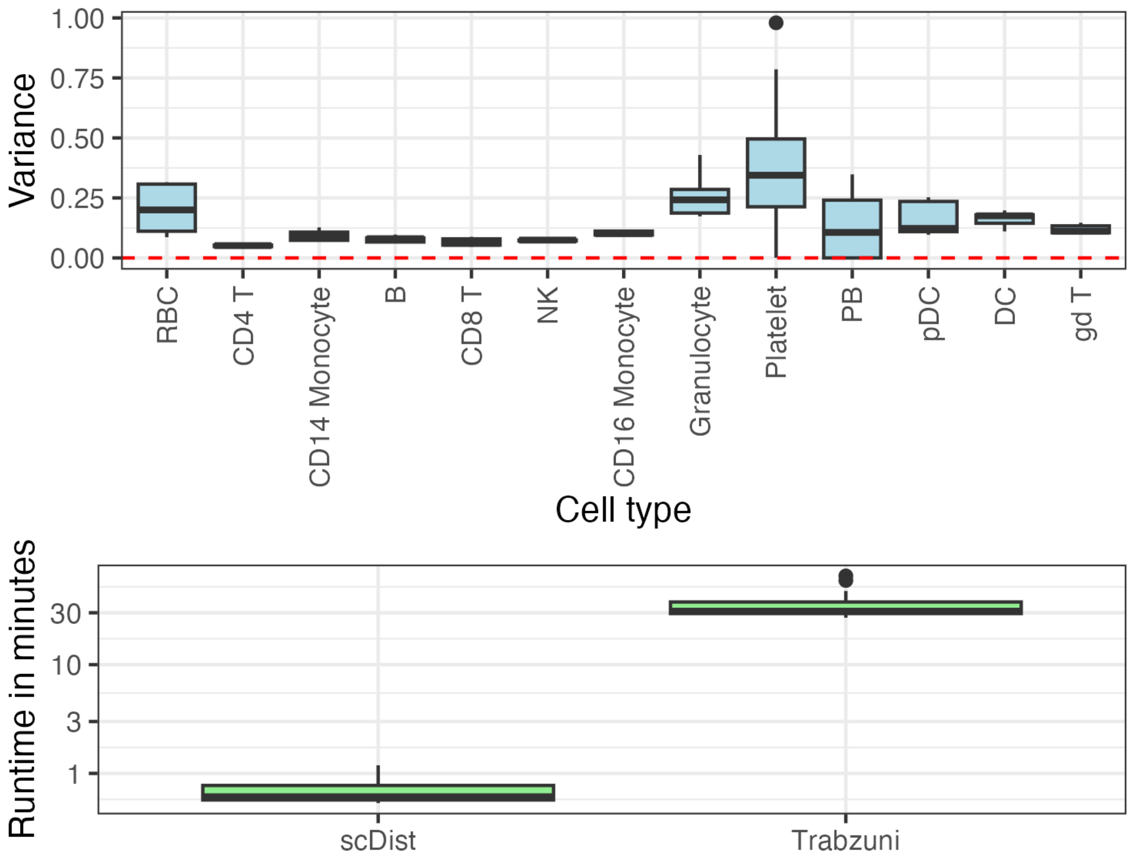

Figure S28: Top: Repeating the analysis of Figure 1 using the method of (4) to measure cell type perturbation (C). The dashed line represents the expected zero perturbation. Bottom: The runtime in minutes of scDist and the Trabzuni method across the 20 repetitions. Source data are provided as a Source Data file.

References

[1] Satija, R., Farrell, J. A., Gennert, D., Schier, A. F. & Regev, A. Spatial reconstruction of single-cell gene expression data. *Nature biotechnology* **33**, 495–502 (2015).

[2] Yu, L., Zhang, J., Brock, G. & Fernandez, S. Fully moderated t-statistic in linear modeling of mixed effects for differential expression analysis. *BMC bioinformatics* **20**, 1–9 (2019).

[3] Ritchie, M. E. *et al.* limma powers differential expression analyses for rna-sequencing and microarray studies. *Nucleic acids research* **43**, e47–e47 (2015).

[4] Trabzuni, D., (UKBEC), U. K. B. E. C. & Thomson, P. C. Analysis of gene expression data using a linear mixed model/finite mixture model approach: application to regional differences in the human brain. *Bioinformatics* **30**, 1555–1561 (2014).

[5] Korsunsky, I. *et al.* Fast, sensitive and accurate integration of single-cell data with harmony. *Nature methods* **16**, 1289–1296 (2019).

[6] Crowell, H. L. *et al.* Muscat detects subpopulation-specific state transitions from multi-sample multi-condition single-cell transcriptomics data. *Nature communications* **11**, 6077 (2020).

[7] Wilk, A. J. *et al.* A single-cell atlas of the peripheral immune response in patients with severe covid-19. *Nature medicine* **26**, 1070–1076 (2020).

[8] Bates, D., Mächler, M., Bolker, B. & Walker, S. Fitting linear mixed-effects models using lme4. *Journal of Statistical Software* **67**, 1–48 (2015).

[9] Yuen, K. C. *et al.* High systemic and tumor-associated il-8 correlates with reduced clinical benefit of pd-l1 blockade. *Nature medicine* **26**, 693–698 (2020).

[10] Yost, K. E. *et al.* Clonal replacement of tumor-specific t cells following pd-1 blockade. *Nature medicine* **25**, 1251–1259 (2019).

[11] Luoma, A. M. *et al.* Tissue-resident memory and circulating t cells are early responders to pre-surgical cancer immunotherapy. *Cell* **185**, 2918–2935 (2022).

[12] Sade-Feldman, M. *et al.* Defining t cell states associated with response to checkpoint immunotherapy in melanoma. *Cell* **175**, 998–1013 (2018).
